# Impact of two consecutive COVID-19 outbreaks on the physical fitness in college students: a five-year longitudinal study

**DOI:** 10.1186/s12889-026-27559-y

**Published:** 2026-04-28

**Authors:** Qianqian Jiao, Jianzhong Sun, Xi Zhang, Yaru Guo, Hao Xue, Feng Li

**Affiliations:** 1 Department of Sports Science, Wenzhou Medical University, Wenzhou, Zhejiang China; 2 School of Physical Education, Chizhou University, Chizhou, Anhui China; 3 College of Sports Industry and Leisure, Nanjing Sport University, Nanjing, China

**Keywords:** Twice COVID-19 epidemics, College students, Physical fitness, Profound impact

## Abstract

**Background:**

The COVID-19 pandemic has profoundly affected global public health, yet the long-term and compounded effects of consecutive outbreaks on university students’ physical fitness remain underexplored. This study investigates the multifaceted impact of two consecutive COVID-19 outbreaks—the initial global pandemic in late 2019 and a localized campus outbreak in late 2022—on college students’ physical fitness over five year.

**Methods:**

A longitudinal analysis was conducted using five consecutive years (2019–2023) of physical fitness test data from 74,394 undergraduate students at Chizhou University. Fitness indicators included body mass index (BMI), vital capacity, 50-m dash, standing long jump, sit-and-reach, pull-ups (male)/sit-ups (female), and 1000-m (male)/800-m (female) run. Non-parametric tests examined annual changes stratified by gender and BMI category.

**Results:**

The two outbreaks affected fitness components differently. Overweight and obesity rates increased significantly, especially in 2023. Endurance running and 50-m dash performance declined during both outbreak periods. Total fitness scores decreased during outbreaks but were significantly higher in 2023 than in 2020. Conversely, vital capacity, sit-and-reach, and pull-ups/sit-ups markedly improved in 2023. Gender-specific trends appeared across multiple indicators. Underweight and obesity students showed gradual total score improvements over time, while normal-weight and overweight groups experienced initial declines followed by recovery.

**Conclusions:**

Two consecutive COVID-19 outbreaks exerted complex and divergent effects on college students’ physical fitness. Body weight and sprint performance were negatively impacted, but the second outbreak saw improvements in several indicators—likely due to more structured online physical education and increased exercise awareness. These findings underscore the need for adaptive, resilient physical education strategies and targeted health interventions in response to public health crises.

**Supplementary Information:**

The online version contains supplementary material available at 10.1186/s12889-026-27559-y.

## Background

Since its emergence in late 2019, COVID-19 has spread rapidly, profoundly effecting global economic, social, and health systems [1–3]. Public health measures implemented to curb transmission—such as social isolation, campus closures, and online learning—substantially altered university students’ daily habits [4–8], reducing opportunities for physical activity and increasing sedentary behavior [9, 10]. These changes compromised students’ physical fitness [11, 12] and exacerbated mental health issues, further undermining student well-being [9, 13].

The initial outbreak in 2019 prompted nationwide containment measures in China, including travel restrictions and routine nucleic acid testing [14–16]. Despite these efforts, subsequent localized clusters emerged [17]. Extensive research has examined student physical health after the first outbreak, focusing on overall fitness changes [2, 3, 18, 19], overweight and obesity [20, 21], physical activity patterns [22, 23], psychological distress [1, 3, 24], and academic stress [4–25]. However, research on the second wave remains scarce. Existing international literature primarily addresses informal caregivers health and clinical manifestations in COVID-19 patients [13, 26–28], with no studies have investigated physical fitness indicators. Within China, no studies have yet examined the impacts of a second localized outbreak, nor the compound effects of two consecutive outbreaks on university students.

COVID-19 has significantly impaired physical and mental health globally [2, 29], making the promotion of physical exercise crucial to counteracting long-term negative effects [19]. Chizhou University experienced two distinct COVID-19 outbreaks: the initial global pandemic in late 2019 and a localized campus outbreak in late 2022 [30]. This study systematically evaluates the multidimensional effects of these two outbreaks by analyzing five consecutive years (2019–2023) of physical fitness test data from all undergraduate students. Specifically, it aims to: ① compare the magnitude and direction of changes in physical fitness indicators before and after the two epidemics; ② identify differential response patterns across indicators (weight status, aerobic endurance, strength, flexibility and speed); and ③ explore heterogeneity across gender and BMI subgroups to inform targeted public health and physical education interventions.

## Methods

### Participants and data source

This study investigated all undergraduate students at Chizhou University with complete physical fitness test data from 2019 to 2023. The annual number of eligible participants is detailed in Table 1 (participant section). A cluster longitudinal design was emplyed without sampling. Missing values or logical errors in the data were excluded or processed before inclusion in the analysis. Informed consent was obtained from all participating students. For those under 17 years of age, written authorization was provided by their guardians. The study protocol was approved by the Ethics Committee of Chizhou University (Approval Nos. SX2019CZU024 and SX2021CZU029), and strictly adhered to the Declaration of Helsinki. To ensure confidentiality, all participant names and personal identifiers were encrypted.

**Table 1:** Characteristics (N+%) of participants used in this study

PF indicators	2019	2020	2021	2022	2023
Age(year)	male: 58.3%	male: 57.0%	male: 55.1%	male: 52.3%	male: 51.0%
≤ 17	218(1.5)	231(1.5)	212(1.4)	181(1.1)	182(1.1)
18	1,750(12.6)	1,784(12.0)	1,852(13.0)	2,045(13.3)	2,345(14.3)
19	2,992(21.7)	2,965(20.0)	2,892(20.4)	3,300(21.5)	3,766(23.0)
20	3,168(22.9)	3,430(23.1)	3,288(23.2)	3,526(23.0)	3,691(22.6)
21	2,931(21.2)	3,247(21.9)	3,329(23.4)	3,433(22.4)	3,435(21.0)
22	1,796(13.0)	2,026(13.6)	1,836(12.9)	2,047(13.3)	1,984(12.1)
23	665(4.8)	827(5.5)	574(4.0)	636(4.1)	694(4.2)
≥ 24	261(1.8)	290(1.9)	186(1.3)	155(1.0)	224(1.3)
Total participant	13,781	14,800	14,169	15,323	16,321
Vital capacity (ml)	*P*1 < 0.001^#^, *P*2 = 0.53, *P*3 < 0.001^#^, *P*4 < 0.001^#^				
Excellent (≥ 4800 for male, ≥ 3000 for female)	591(4.2)	871(5.8)	13,48(9.5)	3,541(23.1)	5,868(35.9)
Good (≥ 4300 and < 4800 for male, ≥ 3000 and < 3300 for female)	1,051(7.6)	1,613(10.8)	1,213(8.5)	2,759(18.0)	3,201(19.6)
Pass (≥ 3100 and < 4300 for male, ≥ 2000 and < 3000 for female)	10,124(73.4)	11,351(76.6)	10,795(76.1)	8,347(54.4)	6,919(42.3)
Fail (< 3100 for male, < 2000 for female)	2015(14.6)	965(6.5)	813(5.7)	676(4.4)	333(2.0)
Standing long jump (cm)	*P*1 = 0.12, *P*2 < 0.001^#^, *P*3 < 0.001^#^, *P*4 < 0.001^#^				
Excellent (≥ 263 for male, ≥ 195 for female)	681(4.9)	509(3.4)	1,334(9.4)	1,734(11.3)	2,230(13.6)
Good (≥ 248 and < 263 for male, ≥ 181 and < 195 for female)	1,595(11.5)	1,770(11.9)	1,488(10.5)	2,336(15.2)	2,733(16.7)
Pass (≥ 208 and < 248 for male, ≥ 151 and < 181 for female)	9,621(69.8)	11,651(78.7)	10,467(73.8)	10,468(68.3)	9,912(60.7)
Fail (< 208 for male, < 151 for female)	1,884(13.6)	870(5.8)	880(6.2)	785(5.1)	1,446(8.8)
50-m dash (s)	*P*1 < 0.001^#^, *P*2 < 0.001^#^, *P*3 < 0.001^#^, *P*4 = 1.0				
Excellent (≤ 6.9 for male, ≤ 7.7 for female)	1,382(10.0)	988(6.6)	1,992(14.0)	768(5.0)	1,478(9.0)
Good (≤ 7.1 and > 6.9 for male, ≤ 8.3 and > 7.7 for female)	1,365(9.9)	1,288(8.7)	1,451(10.2)	1,270(8.2)	1,217(7.4)
Pass (≤ 9.1 and > 7.1 for male, ≤ 10.3 and > 8.3 for female)	9,940(72.1)	12,195(82.3)	10,153(71.6)	11,811(77.0)	11,178(68.4)
Fail (> 9.1 for male, > 10.3 for female)	1094(7.9)	329(2.2)	573(4.0)	1474(9.6)	2448(14.9)
Total score	*P*1 < 0.001^#^, *P*2 < 0.001^#^, *P*3 < 0.001^#^, *P*4 = 0.58				
Excellent (≥ 90)	17(0.1)	50(0.3)	456(3.2)	351(2.2)	520(1.3)
Good (≥ 80 and < 90)	986(7.1)	764(5.1)	1,300(9.1)	4,301(28.0)	4,460(27.3)
Pass (≥ 60 and < 80)	11,202(81.2)	12,395(83.7)	11,645(82.1)	9,570(62.4)	9,814(60.1)
Fail (< 60)	1,576(11.4)	1,591(10.7)	768(5.4)	1,101(7.1)	1,527(9.3)

*cm*,* centimeter; ml*,* milliliter; s*,* second*

*P*1, *P*2, *P*3, *P*4, mean the *p*-values of Kruskal-Wallis H test between 2019 and 2020, 2020–2021, 2021–2022, and 2022–2023.^#^*P* < 0.001

The data preceding the parentheses are the specific number of participants, while the data in parentheses are the percentages (%)

### Patient and public involvement

Patients and/or the public were not involved in the design, conduct, reporting or dissemination plans of this research. Findings of this research will be disseminated through scientific publications and conference presentations to inform public health practice and future policies aimed at improving college students’ physical fitness.

### The influence of twice COVID-19 epidemics

The first COVID-19 cases were reported in Wuhan, China, in December 2019. For Chizhou University, the initial outbreak occurred during the winter vacation. All students had returned to their hometowns and were thus largely unaffected at that time. Although not directly affected by the epidemic, college students experienced a significant decline in physical activity during the Spring Festival holiday. As the most important traditional holiday in China, it is typically marked by family gatherings, festive eating, drinking, and leisure activities, leaving little time for moderate-to-vigorous physical exercise—often resulting in noticeable weight gain.

The Spring 2020 semester, starting in February, was severely disrupted. Students were prohibited from returning to campus without authorization. Online classes began on February 17, 2020, with staggered returns commencing in May 2020. Returning students required negative nucleic acid test, mask-wearing, temperature checks, and a two-week campus lockdown. During this period, public physical education courses were delivered online, emphasizing theoretical knowledge and movement demonstrations. Students were encouraged to independently meet the prescribed exercise volume and duration. As an unprecedented public health event, the pandemic caused immense psychological stress for teachers and students alike, leading to a marked decline in physical activity, with both the volume and intensity of exercise falling well below adequate levels.

In November 2022, nine new COVID-19 cases were reported in Guichi District, Chizhou City, including one asymptomatic case at Chizhou University. The campus was immediately designated as a high-risk area, prompting stringent measures: daily nucleic acid testing for 20% of students and staff, dormitory quarantine, temperature checks, cancellation of gatherings, and meal delivery by teacher volunteers. Physical education courses returned online, with curricula emphasizing stretching, strength and power training, and Baduanjin exercises. During this period, although students were mostly confined to dormitories, teachers emphasized exercise volume and intensity. Enhanced measures included online check-ins and offline assessments. A designated student in each dormitory recorded activity types (diversified to ensure muscle strength and cardiorespiratory endurance) and duration (for example, a full session of Baduanjin takes 12 min and is practiced in the morning, noon, and evening). Students were encouraged to support each other and maintain a positive mindset. The curriculum was well-structured and diverse, ensuring adequate exercise volume and intensity. Notably, some fitness data from 2022 (such as body weight, endurance, and other physical fitness test results) may reflect the cumulative effects of the 2020–2021 period.

## Measurements

Physical fitness assessment included eight metrics for both male and female students: height, weight, vital capacity, 50-m dash, standing long jump, sit-and-reach, pull-ups (male)/sit-ups (female), and 1000-m run (male)/800-m run (female). Detailed procedures, testing instruments, and calibration methods have been previously described [31] and abbreviated as follows.

All measurements were conducted by trained assessors following standardized protocols per the National Physical Fitness Measurement Standards Manual (NPFMSM). To reduce intra-individual variability, all tests for each participant were completed on the same day in a fixed sequence: indoor assessments (height, weight, vital capacity, sit-and-reach, and female sit-ups) after a brief warm-up; outdoor tests (standing long jump, male pull-ups, and 50-m dash); and finally, the endurance run (1000-m for males, 800-m for females) preceded by a full dynamic warm-up.

Height was recorded to the nearest 0.1 cm using a portable stadiometer; weight to the nearest 0.1 kg with a calibrated scale, with participants in light clothing and barefoot. Vital capacity was assessed using a digital spirometer (TSN200-FH, Tishineng Sports), with the highest value from two trials (in milliliters, rounded to integers) retained. The 50-m dash was timed to 0.1 s using an automated system (TSN200-WP, Tishineng Sports), with each participant completing one trial. Standing long jump was measured on dedicated mats (TSN200-TY, Tishineng Sports), with the best distance from two attempts (in centimeters) recorded. Sit-and-reach was assessed using a flexibility tester (TSN200-TQ, Tishineng Sports), with the farthest reach from two trials recorded. Upper body strength was evaluated via pull-ups for males (TSN200-YT, Tishineng Sports) using a standard overhand grip on a horizontal bar, and one-minute sit-ups for females (TSN200-YW, Tishineng Sports) with knees bent at 90°, each tested once. Endurance capacity was measured using a long-distance running timing system (TSN200-CP, Tishineng Sports), with run times recorded to 0.1 s. All testing instruments were Bluetooth-enabled for automatic data transmission.

### Statistical analysis

All physical fitness indicators were classified and scored in accordance with the National Student Physical Health Standard (2014 Edition). Missing and illogical values were processed or excluded. The approach involved identifying outliers based on biologically plausible ranges, correcting or flagging adjustable values, and excluding clearly erroneous data (e.g., due to recording errors or equipment malfunction) to ensure internal consistency and a reasonable biological distribution.

Normality and homogeneity of variance were assessed using SPSS 25.0. First, Levene’s test and the Kolmogorov–Smirnov test was used to analyze the homogeneity of variance, followed by the Kolmogorov–Smirnov test to assess normality. Data were non-normally distributed with unequal variances. Categorical data are presented as counts (percentages), continuous data as medians (interquartile ranges). The non-parametric Kruskal-Wallis H test was used for overall score comparisons, with Bonferroni post-hoc tests for group comparisons by gender and BMI categories.

## Results

A total of 74,394 university students participated, with annual eligible participants from 2019 to 2023 numbering 13,781, 14,800, 14,169, 15,323, and 16,321, respectively. Participants were predominantly aged 18–23 years. Table 1 summarizes annual age statistics and the distribution of performance grades (excellent, good, pass, and fail) for vital capacity, standing long jump, 50-m, and total fitness score. Supplementary Table S1 provides detailed analyses for BMI, sit-and-reach, pull-ups/sit-ups, and 1000/800-m run.

For all fitness metrics except BMI, the majority of students (60.1%~83.7%) received a “Pass” grade, with distribution following the pattern: Pass > Fail > Good > Excellent. For BMI, most students (63.7%~77.2%) were within the normal weight range, followed by overweight, underweight, and obesity. Table 1 and S1 present year-to-year statistical comparisons. Table 1 indicates no significant difference in vital capacity between 2020 and 2021, standing Long Jump between 2019 and 2020, or 50-m dash and total score between 2022 and 2023. Supplementary Table S1 shows no significant difference in BMI between 2019 and 2021, or in 1000/800-m between 2019 and 2022. All other year-to-year comparisons were statistically significant (*P* < 0.05), wtih most at *P* < 0.001.

**Fig. 1:**
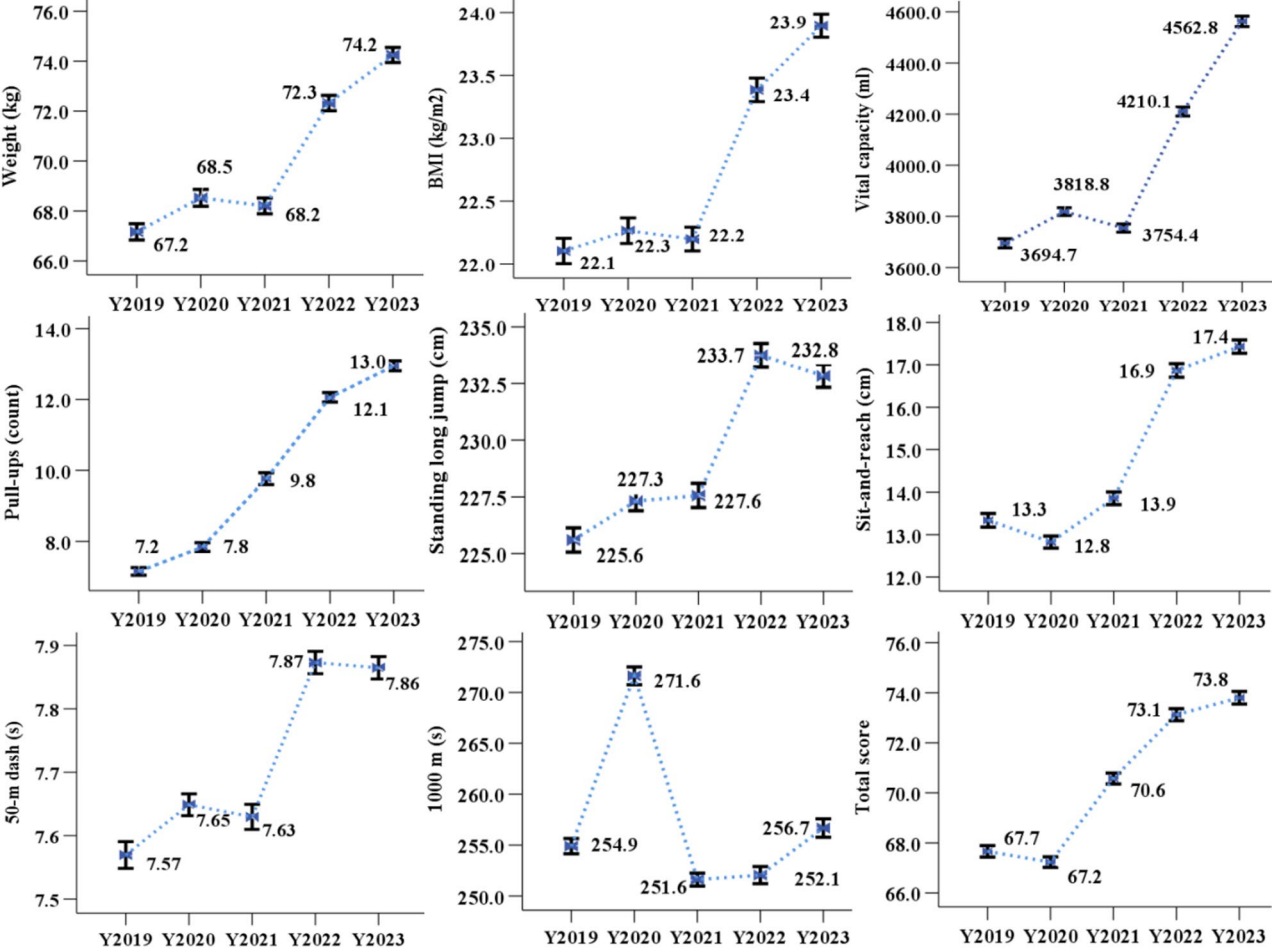
The mean and 95% CI of items of the physical fitness indexes on male from 2019 to 2023. For male, the graph plots year on the x-axis against the values of physical fitness indicators on the y-axis. Data points (blue) represent the mean values for each year, with error bars (parallel black lines) denoting the 95% confidence interval. The 5-year trend for each indicator is depicted by a blue dashed line

**Fig. 2:**
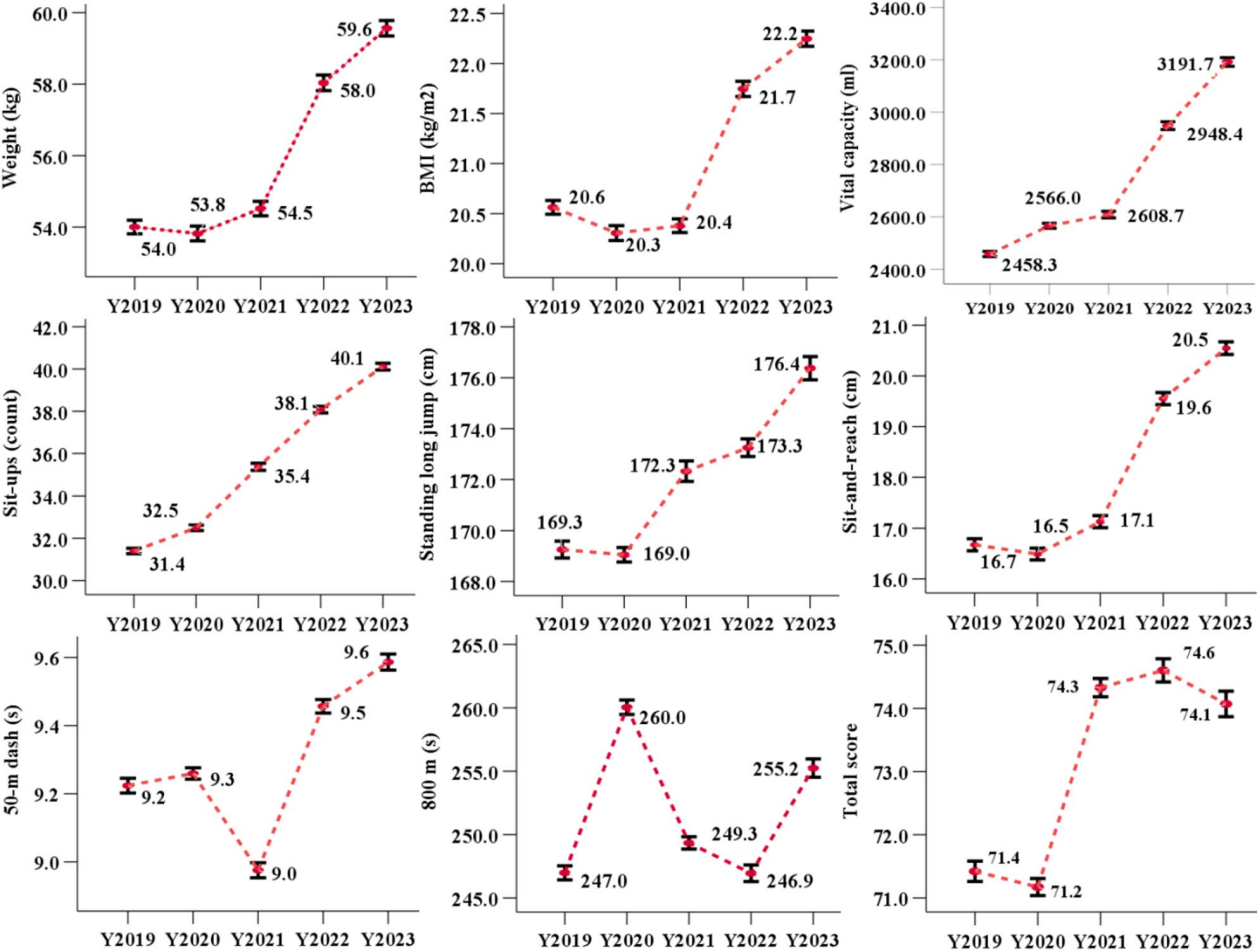
The mean and 95% CI of items of the physical fitness indexes on female from 2019 to 2023. For female, the graph plots year on the x-axis against the values of physical fitness indicators on the y-axis. Data points (red) represent the mean values for each year, with error bars (parallel black lines) denoting the 95% confidence interval. The 5-year trend for each indicator is depicted by a red dashed line

Table 2 and Supplementary Table S2 provide annual median and interquartile range for each fitness metric by gender, with year-to-year comparisons. Together with Figs. 1 and 2, these illustrate five-year trends. Body weight, BMI, vital capacity, and pull-ups/sit-ups generally increased over time for both genders. Significant changes (*P* < 0.001) were observed between 2019 and 2020 for male weight, vital capacity, and pull-ups, and for female weight, BMI, vital capacity, and sit-ups. In contrast, no significant change in male BMI between 2019 and 2020 (*p* = 1.0). In 2020, male weight (67.2 to 68.5 kg), vital capacity (3694.7 to 3818.8 ml), and pull-up performance (7.2 to 7.8 reps) increased significantly compared to 2019. Female weight (54.0 to 53.8 kg) and BMI (20.6 to 20.3 kg/m²) decreased significantly in 2019–2020, while vital capacity (2458.3 to 2566.0 ml) and sit-up performance (31.4 to 32.5 reps) increased significantly. Between 2022 and 2023, both genders also showed highly significant increases (*P* < 0.001) in weight, BMI, vital capacity, and pull-ups/sit-ups.

**Table 2:** Median (interquartile range) and difference comparison of physical fitness indicators from 2019 to 2023 by gender

Gender	Items	2019(A)	2020(B)	2021(C)	2022(D)	2023(E)	A-B	B-C	C-D	D-E
Male	Height (cm)	174.0(8.0)	175.0(7.0)	175.0(7.0)	175.6(8.0)	176.1(8.1)	< 0.001^#^	1	< 0.001^#^	< 0.001^#^
	Weight (kg)	65.0(14.0)	65.0(15.0)	65.0(15.0)	70.2(16.0)	72.1(16.8)	< 0.001^#^	1	< 0.001^#^	< 0.001^#^
	BMI (kg/m^2^)	21.4(4.4)	21.5(4.4)	21.5(4.6)	22.7(4.7)	23.3(4.9)	1	1	< 0.001^#^	< 0.001^#^
	Vital capacity (ml)	3,646(820.8)	3,729(818.5)	3,638(798.0)	4,181(1030.0)	4,485(1175.0)	< 0.001^#^	< 0.001^#^	< 0.001^#^	< 0.001^#^
	Standing long jump (cm)	225.0(30.0)	225.0(22.0)	225.0(27.0)	230.0(35.0)	231.0(35.0)	0.01*	1	< 0.001^#^	1
	Sit-and-reach (cm)	13.2(8.5)	12.5(7.5)	14.0(8.0)	17.0(11.0)	17.5(44.0)	< 0.001^#^	< 0.001^#^	< 0.001^#^	< 0.001^#^
	50-m dash (s)	7.5(0.9)	7.5(0.9)	7.6(1.0)	7.8(1.1)	7.9(1.1)	< 0.001^#^	1	< 0.001^#^	0.55
	Pull-ups (count)	7.0(6.0)	7.0(6.0)	10.0(7.0)	12.0(8.0)	12.0(10.0)	< 0.001^#^	< 0.001^#^	< 0.001^#^	< 0.001^#^
	1000 m (s)	252.0(31.0)	268.0(49.0)	246.0(23.0)	247.0(41.0)	251.0(47.0)	< 0.001^#^	< 0.001^#^	1	< 0.001^#^
	Total score	68.3(11.1)	67.5(11.0)	70.4(10.7)	73.4(14.5)	75.2(15.2)	< 0.001^#^	< 0.001^#^	< 0.001*	< 0.001^#^
Female	Height (cm)	162.0(6.0)	163.0(6.0)	163.0(7.0)	163.3(7.1)	163.6(7.4)	< 0.001^#^	< 0.001^#^	1	0.03*
	Weight (kg)	53.0(9.3)	52.0(9.0)	53.0(10.0)	56.3(11.4)	58.1(11.5)	< 0.001^#^	< 0.001^#^	< 0.001^#^	< 0.001^#^
	BMI (kg/m^2^)	20.0(3.1)	19.6(3.1)	19.8(3.2)	21.1(3.8)	21.7(3.9)	< 0.001^#^	< 0.001^#^	< 0.001^#^	< 0.001^#^
	Vital capacity (ml)	2,420(548.0)	2,518(582.8)	2,524(681.0)	2,852(909.5)	3,089(911.0)	< 0.001^#^	0.33	< 0.001^#^	< 0.001^#^
	Standing long jump (cm)	170.0(20.0)	170.0(20.0)	170.0(20.0)	170.0(21.0)	175.0(28.0)	1	< 0.001^#^	< 0.001^#^	< 0.001^#^
	Sit-and-reach (cm)	17.0(7.0)	17.0(7.0)	17.0(7.0)	20.0(7.5)	21.0(7.9)	0.08	< 0.001^#^	< 0.001^#^	< 0.001^#^
	50-m dash (s)	9.2(1.2)	9.3(1.0)	9.0(1.2)	9.5(1.1)	9.7(1.4)	< 0.001^#^	< 0.001^#^	< 0.001^#^	< 0.001^#^
	Sit-ups (count)	31.0(7.0)	32.0(7.0)	35.0(8.0)	37.0(9.0)	40.0(10.0)	< 0.001^#^	< 0.001^#^	< 0.001^#^	< 0.001^#^
	800 m (s)	244.0(27.0)	257.0(33.0)	249.0(24.0)	245.0(29.0)	252.0(34.0)	< 0.001^#^	< 0.001^#^	< 0.001^#^	< 0.001^#^
	Total score	72.8(8.4)	72.0(7.4)	74.4(6.4)	74.8(11.1)	74.8(11.7)	< 0.001^#^	< 0.001^#^	0.08	0.02*

A-B means the *P*-values of Kruskal-Wallis H test between 2019–2020 (for example); ^*^P < 0.05, ^#^*P* < 0.001

The data preceding the parentheses are the median values of each physical fitness indicator, while the data in parentheses are the interquartile ranges

Sit-and-reach for both genders, total score for males, and standing long jump for females showed an initial decline followed by an increase, with the turning point in 2020. Male sit-and-reach decreased significantly from 2019 to 2020 (13.3 to 12.8 cm, *P* < 0.001), the improved from 2022 to 2023 (16.9 to 17.4 cm for males;19.6 to 20.5 cm for females). Female standing long jump also improved significantly from 2022 to 2023 (173.7 to 176.4 cm). Male total fitness score decreased from 2019 to 2020 (67.7 to 67.2) and increased from 2022 to 2023 (73.1 to 73.8, both *P* < 0.001). In contrast, no significant changes (*P* > 0.05) were found from 2019 to 2020 for female standing long jump (169.3 to 169.0 cm) or sit-and-reach (16.7 to 16.5 cm).

Male 50-m dash and standing long jump exhibited initial improvement followed by decline. Both changed significant (*P* < 0.001) between 2019 and 2020. The 50-m time increased from 7.57 to 7.65 s, while standing long jump increased from 225.6 to 227.3 cm. In contrast, no significant differences (*P* > 0.05) were observed for these two metrics between 2022 and 2023.

Male 1000-m performance showed an irregular trend. Significant declines (*P* < 0.001) were observed between 2019 and 2020 (from 254.9 to 271.6 s) and between 2022 and 2023 (from 252.1 to 256.7 s). Female 50-m, 800-m, and total fitness score also displayed irregular trends. All three metrics declined significantly (*P* < 0.001) in both the 2019–2020 and 2022–2023 periods. Specifically, female 50-m times increased from 9.2 to 9.3 s (2019–2020) and from 9.5 to 9.6 s (2022–2023). The 800-m times increased from 247.0 to 260.0 s and from 246.9 to 255.2 s, respectively. Total fitness scores decreased from 71.4 to 71.2 points and from 74.6 to 74.1 points over the same intervals.

**Fig. 3:**
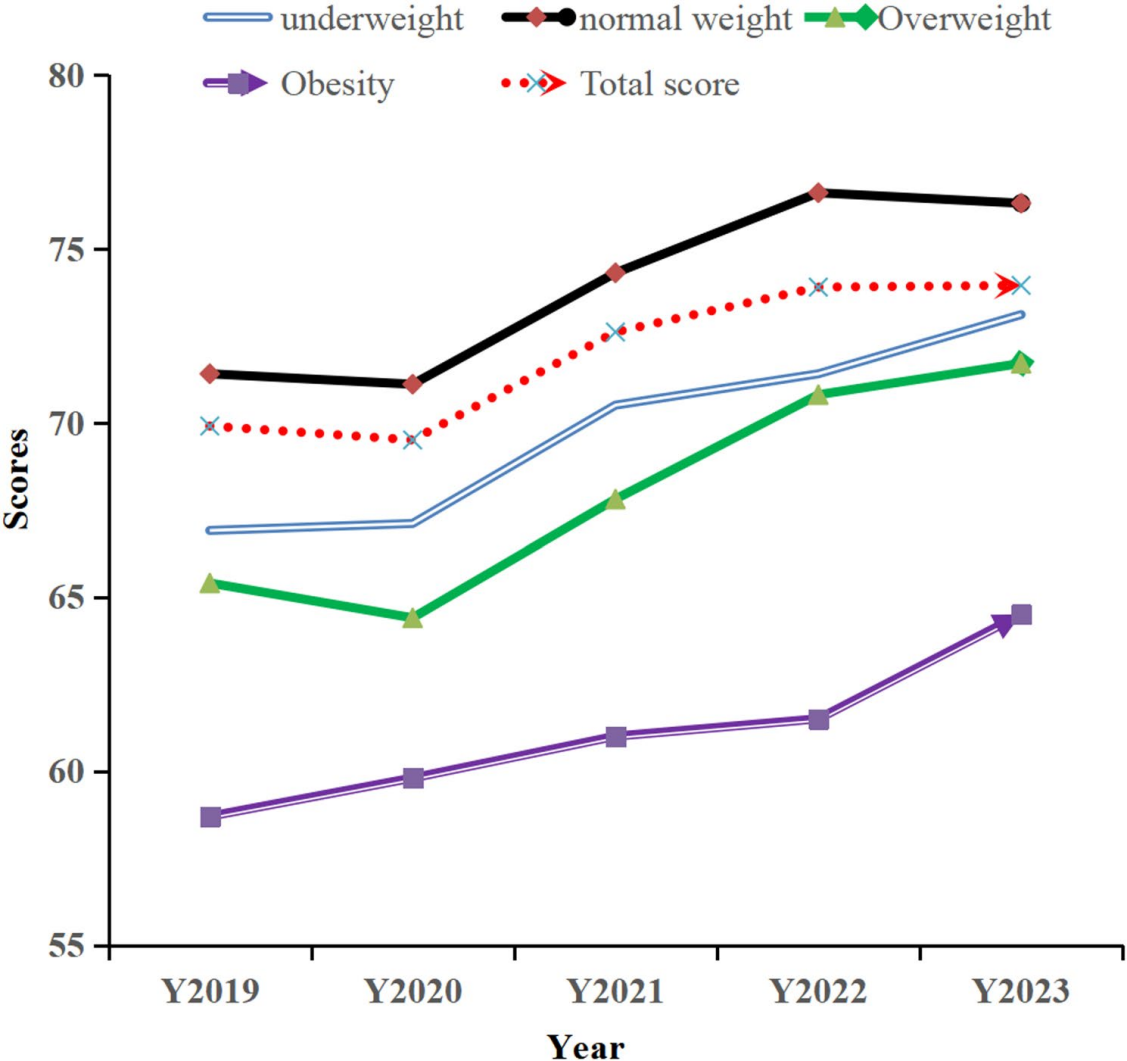
The trend chart of the total score of BMI four grades. The x-axis represents the year, and the y-axis represents the physical fitness test score. The red dashed line traces the overall trend for the entire student cohort. Trends for specific weight categories—normal weight (black), underweight (blue), overweight (green), and obesity (purple)—are shown in the corresponding colored lines

Figure 3 and Supplementary Table S3 illustrate five-year trends in total fitness scores across BMI categories, with year-to-year comparisons in Table 3. Underweight and obese groups exhibited a increasing trend year by year in their scores, with no significant change between 2019 and 2020 but significant increases from 2022 to 2023 (*P* < 0.05; underweight: 71.4 to 73.1; obese: 61.5 to 64.5). Total score, normal weight, and overweight groups showed an initial decline followed by increase, with significant decreases from 2019 to 2020 (*P* < 0.001; total score: 69.9 to 69.5; normal weight: 71.4 to 71.1; overweight: 65.4 to 64.4). From 2022 to 2023, only the overweight group showed a significant score increase (*P* < 0.05, 70.8 to 71.7). No significant changes (*P* > 0.05) were observed for the total score or the normal weight group during this time.

**Table 3:** Comparison of the differences of total physical test scores of BMI four grades

BMI	A-B	B-C	C-D	D-E	B-D	B-E	C-A	C-E	D-A	E-A
underweight	1	< 0.001^#^	0.12	0.01*	< 0.001^#^	< 0.001^#^	< 0.001^#^	< 0.001^#^	< 0.001^#^	< 0.001^#^
normal	< 0.001^#^	< 0.001^#^	< 0.001^#^	0.81	< 0.001^#^	< 0.001^#^	< 0.001^#^	< 0.001^#^	< 0.001^#^	< 0.001^#^
overweight	< 0.001^#^	0*	< 0.001^#^	0.004*	< 0.001^#^	< 0.001^#^	< 0.001^#^	< 0.001^#^	< 0.001^#^	< 0.001^#^
obesity	1	0.19	1	< 0.001^#^	0.003*	< 0.001^#^	0.003*	< 0.001^#^	< 0.001^#^	< 0.001^#^
Total score	< 0.001^#^	< 0.001^#^	< 0.001^#^	0.58	< 0.001^#^	< 0.001^#^	< 0.001^#^	< 0.001^#^	< 0.001^#^	< 0.001^#^

A-Y2019, B-Y2020, C-Y2021, D-Y2022, E-Y2023; ^*^P < 0.05, ^#^*P* < 0.001

The data in the table are the *P*-values for the comparisons of differences between the two years

**Fig. 4:**
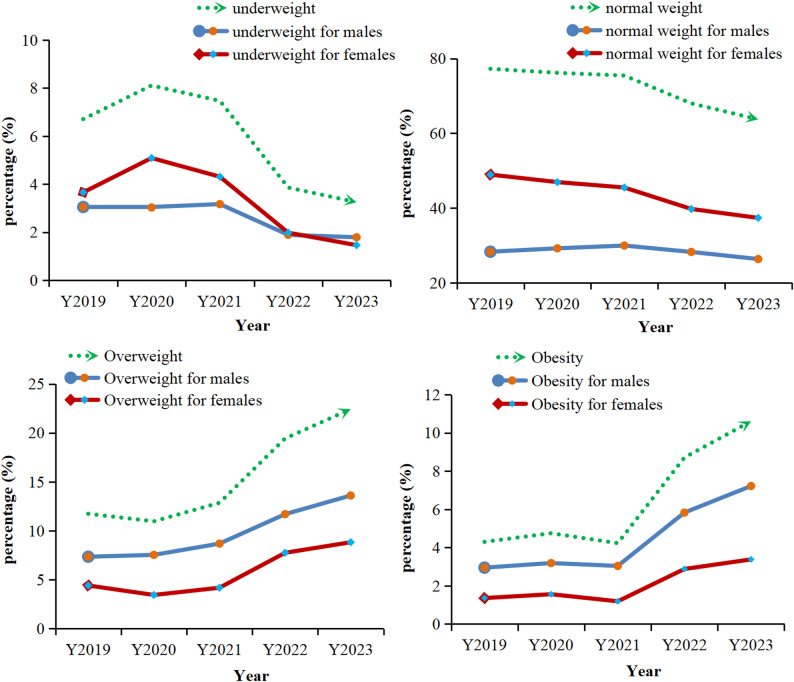
Trend chart of the percentage (%) in four grades of BMI from 2019–2023. Figure 4 shows the percentage of students in each BMI category over time (year on the x-axis). The overall trend for the entire cohort is shown by the green dashed line. The blue and red solid lines trace the corresponding percentages for male and female subpopulations, respectively

Figure 4 and Supplementary Table S4 show trends in BMI category the proportions. The overall trends for all BMI categories, as well as the separate trends for male and female students, were largely consistent. Underweight proportion initially increased then decreased (6.7%→8.1%→7.5%→3.8%→3.3%). Normal weight generally declined (77.3%→76.2%→76.5%→68.0%→63.7%). In contrast, overweight (11.7%→10.9%→12.8%→19.4%→22.4%) and obese (4.3%→4.7%→4.2%→8.7%→10.6%) showed upward trends. Notably, the proportion of students with obesity decreased noticeably in 2021 (4.7% to 4.2%). Between 2019 and 2020, underweight (more markedly in females: 3.7%→5.1%) and obesity (males: 2.9%→3.2%; females: 1.4%→1.6%) increased significantly. Meanwhile, normal weight (more markedly in females: 48.9%→46.9%) and overweight (more markedly in females: 4.4%→3.4%) declined. From 2022 to 2023, underweight (more markedly in females: 1.9%→1.5%) and normal weight (more markedly in females: 39.7% → 37.4%) continued to decline, while overweight (more markedly in males: 11.7%→13.6%) and obese (more markedly in males: 5.8%→7.2%) increased.

## Discussion

This study found that most physical fitness indicators (except BMI) clustered near the passing threshold, with over 50.0% of students in this category. The general trend observed was: pass > fail > good > excellent, consistent with our previous research [32]. Hao et al. [1] reported declines physical fitness following COVID-19 infection, which may partly explain the prevalence of passing scores [1].

BMI increase significantly from 2021 to 2023, with the sharpest rise in 2022, indicating considerable weight gain during the pandemic [3, 9, 29, 33]. Cai et al. [34] observed rising BMI (19.2 to 20.5 kg/m²) and overweight/obesity among Chinese adolescents from 2004 to 2015 [34]. Similarly, studies by Xia et al. [35] and Wang et al. [5] documented reduced physical activity and increased overweight/obesity among university students during the first pandemic wave in 2019 [5, 35]. These changes were also associated with mental health issues. These patterns suggest a persistent upward BMI trend among younger generations in China, with worsening overweight and obesity problems, driven by improved living standards and limited nutritional knowledge. For university students, the first outbreak had limited direct impact on BMI, but effects were lagging behind. The second outbreak, coupled with prolonged sedentary behavior, insufficient exercise, confined campus life, and academic pressure, became key drivers of weight gain [10, 36, 37].

From 2019 to 2020, the proportions of underweight and obese students increased significantly, while normal-weight and overweight proportions declined. From 2022 to 2023, overweight and obese groups grew markedly, while underweight and normal-weight groups continued to decrease. Over the five years spanning two pandemic phases, underweight and normal-weight individuals declined from 2020, while overweight and obesity rates rose from 2020, with a sharper increase after 2021. Chen et al. [38] reported increasing overweight (14.9%) and obesity (5.3%) among medical students in Anhui between 2014 and 2016 [38]. Sun et al. [39] found overweight and obesity rates of 28.0% and 12.7% among students at Wannan Medical College during the first pandemic [39]. These findings indicate that weight gain among university students is a long-standing issue, with successive outbreaks contributing to continued increases. Studies by Chen et al. [40] and Ting et al. [20] showed overweight and obesity rates among Chinese adults in 2019 and 2022 were 34.8% vs. 14.1% and 34.3% vs. 11.2%, respectively [20, 40]. Talukdar et al. [41] projected an average annual obesity growth rate of 2.5% from 2019 to 2024 [41]. These findings highlight overweight and obesity as critical concerns across all age groups, necessitating attention from national and institutional authorities. Students should be encouraged to engage in more physical activity during college [42, 43] and develop lifelong exercise habits [44, 45].

Vital capacity increased significantly from 2021 to 2023 for both genders, aligning with findings by Xia et al. [35], Zhou et al. [46], and Sun et al. [39, 35, 39, 46]. Although the first outbreak disrupted physical activity, by second outbreak in 2022, teachers and students had prior pandemic experience, placing greater emphasis on physical exercise as a key countermeasure, contributing to enhanced cardiopulmonary and respiratory fitness. Pull-up and sit-up performance improved significantly over the five years, with greater gains during the second outbreak in 2022. Zhou et al. [46] reported similar improvements among male middle school students in Fujian in 2020 [46]. Conversely, Feng et al. [47] and Ambroży et al. [48] found declines during the first lockdown ( pull-up performance decreased by 23.9% among Tsinghua University students and by 12.7% among Polish university students) [47, 48], likely due to differences in physical education emphasis and online curricula design. Standing long jump performance did not change significantly during the first pandemic period in 2019 but improved in males during the second outbreak in 2022, consistent with Xia et al. (2021) [35]. These exercises are less space-dependent and by 2022, online physical education curricula became more structured, incorporating strength and power exercises.

Sit-and-reach performance declined in 2019–2020 but improved significantly in 2022, contrasting with Xia et al. [35] and Zhou et al. [46, 35, 46]. The first COVID-19 outbreak in 2019 negatively affected the flexibility of the students in this study. This may reflect insufficient self-awareness and physical education requirements duing the first outbreak. By 2022, online curricula emphasized stretching exercises, Baduanjin, as well as strength and power training. With limited options for jogging as warm-up, dynamic and static stretching exercises were more frequently utilized. These activities significantly contributed to the improvement in students’ flexibility [19]. The 50-m performance declined during both outbreaks, consistent with Zhou et al. [46] but differing from Xia et al. [35, 46]. These findings indicate that both COVID-19 outbreaks negatively affected 50-m performance. As a speed-dependent event requiring outdoor spaces, pandemic restrictions hindered sprint training. Indoor exercises were insufficient to maintain normal fitness, and persistent weight gain also contributed.

Endurance running (1000/800-m) declined significantly in 2020 and slightly further in 2023, consistent with previous studies [35, 39, 46, 48]. Increased infection rates led many individuals to abandon previous exercise habits, resulting in sedentary behavior that adversely affected endurance running performance [24]. Zhai et al. [3] noted a lagged effect of the pandemic on muscular endurance and cardiorespiratory fitness [3]. Although lung capacity improved, home quarantine limited muscular strength maintenance. During the second outbreak, more diverse online courses focused on muscular strength training likely mitigated decline [49]. Total physical fitness score declined during the first outbreak for both genders, consistent with Zhao et al. (2024) [4]. In 2021, total scores increased significantly for both genders. During the second outbreak, male scores increased slightly while female scores decreased, differing from Zou et al. (2023) [18]. These results indicate that the first COVID-19 outbreak in 2019 negatively affected total fitness scores, subsequent national policies, institutional efforts, and individual attention to physical exercise contributed to the overall improvement, aligning with Yun et al. (2023) [22].

In summary, under the influence of two COVID-19 epidemics, some physical fitness indicators (vital capacity, pull-ups/sit-ups, and sit-and-reach) showed significant improvement, while others (50-m dash and the 800/1000-m endurance run) declined significantly, alongside rising overweight/obesity rates. This fragmented pattern reflects the complexity of fitness changes during the pandemic. Strength and flexibility can be maintained or improved under constrained conditions through targeted training, whereas weight control and cardiorespiratory endurance rely heavily on sustained, sufficient, and unrestricted aerobic exercise. Despite exercise being an educational priority, its implementation was limited by objective conditions, unable to offset cumulative weight-gain factors.

## Limitations of this study

This study has several limitations. First, data were from a single university over five year, limiting generalizability. Second, limited studies on populations exposed to two consecutive COVID-19 outbreaks and long-term lag effects hampers direct comparisons. Third, the study could not fully control for all external variables (e.g., national physical activity policies, mental health fluctuations, online physical education implementation, nutritional and sleep status), which may have introduced bias. Future research should incorporate broader covariates and use multicenter data with mixed-effects models to better account for confounders.

### Policy and practical implications

### Translation of online physical education experience

The optimized online physical education model from the second epidemic should be integrated into regular blended teaching, and emergency plans for public health emergencies should be established.

### Gender-sensitive perspective

Physical education curricula and health interventions should account for gender differences and develop targeted strategies.

### Support for enabling conditions

Simply “emphasizing exercise” is insufficient to address physical fitness challenges under special circumstances; substantive support in terms of facilities, equipment, time, and guidance is needed.

### Establishment of early warning and evaluation mechanisms

Longitudinal monitoring data should be used to develop an early warning system for student physical health, enabling dynamic assessment of the long-term effects of public health emergencies.

## Conclusions

The consecutive COVID-19 outbreaks significantly impacted university students’ physical fitness. Most students’ fitness scores clustered near the passing threshold. Body weight increased, with overweight and obesity rate rising sharply during the second outbreak (2022–2023). Vital capacity, sit-and-reach, pull-ups, and sit-ups improved markedly in 2023, while endurance running and the 50-m dash declined during both pandemic periods. Total fitness scores decreased during both outbreaks, with a more notable drop in 2019, but were relatively higher during the second outbreak. Trends across various physical fitness indicators differed by gender. Universities should adopt differentiated response strategies. Policies should distinguish between “indicators that can be maintained/improved” and “indicators that are prone to deterioration,” and formulate categorized intervention strategies. Weight management should be prioritized as a core component of health promotion, with attention to lagged effects extending into the recovery period.

## Supplementary Information

Supplementary Material 1.

## Data Availability

Science Data Bank. 10.57760/sciencedb.36908. DOI:10.57760/sciencedb.36908.

## Abbreviations

BMI: body mass index
NPFMSM: National Physical Fitness Measurement Standards Manual
cm: centimeter
ml: milliliter
s: second
kg: kilogram
kg/m^2^: kilogram/meter^2^
