# Supplementary material for: Impact of two consecutive COVID-19 outbreaks on the physical fitness in college students: a five-year longitudinal study
Source: BMC Public Health. 2026 Apr 28;26:1860. doi: 10.1186/s12889-026-27559-y (PMC13262370; doi:10.1186/s12889-026-27559-y)
Supplement: Supplementary file 1 — Supplementary Material 1. [file 12889_2026_27559_MOESM1_ESM.docx]

**Supporting Information for**

**Impact of Two Consecutive COVID-19 Outbreaks on the Physical Fitness in College Students: A Five-Year Longitudinal Study**

Qianqian Jiao^1★,4^,Jianzhong Sun^2★,4^, Xi Zhang^2★,4^, Yaru Guo^2*^, Hao Xue^3*^, Feng Li^1*,5^

^1^Department of Sports Science, Wenzhou Medical University, Wenzhou, Zhejiang, China;

^2^School of Physical Education, Chizhou University, Chizhou, Anhui, China;

^3^College of Sports Industry and Leisure, Nanjing Sport University, Nanjing, China.

^4★^These authors contributed equally to this work.

^5^Lead contact.

*Corresponding author: Yaru Guo, yaru_guo3299@163.com; Hao Xue, xuehao666@126.com; and Feng Li, lifeng@wmu.edu.cn.

| **Table S1 Grading scores of other physical fitness indicators** | | | | | |
| --- | --- | --- | --- | --- | --- |
| **Physical Fitness indicators** | **2019** | **2020** | **2021** | **2022** | **2023** |
| **BMI(kg/m^2^)** | *P*1<0.001^#^, P2<0.001^#^, P3<0.001^#^, P4<0.001^#^, *P*5=1.0, *P*6<0.001^#^, *P*7<0.001^#^, *P*8<0.001^#^, *P*9<0.001^#^*P*10<0.001^#^ | | | | |
| underweight (< 18.5) | 925(6.7) | 1,201(8.1) | 1059(7.4) | 592(3.8) | 531(3.2) |
| normal weight (18.5 ≤ BMI < 23.9) | 10,646(77.2) | 11,274(76.1) | 10,690(75.4) | 10,419(67.9) | 10,399(63.7) |
| overweight (24.0 ≤ BMI < 27.9) | 1,617(11.7) | 1,622(10.9) | 1,821(12.8) | 2,977(19.4) | 3,660(22.4) |
| obesity (≥ 28.0) | 593(4.3) | 703(4.7) | 599(4.2) | 1,335(8.7) | 1,731(10.6) |
| **Sit-and-reach (cm)** | *P*1<0.001^#^, P2<0.001^#^, P3<0.001^#^, P4<0.001^#^, *P*5=0.001*, *P*6<0.001^#^, *P*7<0.001^#^, *P*8<0.001^#^, *P*9<0.001^#^, *P*10<0.001^#^ | | | | |
| Excellent (≥21.3 for male, ≥22.2 for female) | 1,645(11.9) | 1,580(10.6) | 2,206(15.5) | 4,476(29.2) | 5,674(34.7) |
| Good (≥17.7 and <21.3 for male, ≥19 and <22.2 for female) | 2,397(17.3) | 2,319(15.6) | 2,371(16.7) | 3,186(20.7) | 3,326(20.3) |
| Pass (≥3.7 and <17.7 for male, ≥6 and <19 for female) | 9,126(66.2) | 10,653(71.9) | 9,348(65.9) | 7,458(48.6) | 7,107(43.5) |
| Fail (<3.7 for male, <6 for female) | 613(4.4) | 248(1.6) | 24.4(1.7) | 203(1.3) | 214(1.3） |
| **Pull-ups/Sit-ups (Count)** | *P*1<0.001^#^, P2<0.001^#^, P3<0.001^#^, P4<0.001^#^, *P*5<0.001^#^, *P*6<0.001^#^, *P*7<0.001^#^, *P*8<0.001^#^, *P*9<0.001^#^, *P*10<0.001^#^ | | | | |
| Excellent (≥17 for male, ≥52 for female) | 126(0.9) | 364(2.4) | 1,337(9.4) | 1,968(12.8) | 2,991(18.3) |
| Good (≥15 and <17 for male, ≥46 and <52 for female) | 221(1.6) | 3,27(2.2） | 372(2.6) | 1,130(7.3) | 1,813(11.1) |
| Pass (≥10 and <15 for male, ≥26 and <46 for female) | 7,865(57.0) | 8,709(58.8) | 9,307(65.6) | 9,897(64.5) | 9,122(55.8) |
| Fail (<10 for male, <26 for female) | 5,569(40.4) | 5,400(36.4) | 3,153(22.2) | 2,328(15.1) | 2,395(14.6) |
| **1000/800 m (s)** | *P*1<0.001^#^, P2<0.001^#^, P3<0.001^#^, P4<0.001^#^, *P*5<0.001^#^, *P*6=0.32,*P*7<0.001^#^, *P*8<0.001^#^, *P*9<0.001^#^, *P*10<0.001^#^ | | | | |
| Excellent (≤207 for male, ≤210 for female) | 272(2.0) | 158(1.1) | 786(5.5) | 896(5.8) | 788(4.8) |
| Good (≤222 and >207 for male, ≤224 and >210 for female) | 1,038(7.5) | 449(3.0) | 476(3.4) | 1,333(8.7) | 1,468(9.0) |
| Pass (≤272 and >222 for male, ≤274 and >224 for female) | 9,799(71.1) | 8,485(57.3) | 11,166(78.8) | 9,908(64.7) | 9,366(57.4) |
| Fail (>272 for male, >274 for female) | 2,672(19.4) | 5,708(38.6) | 1,741(12.3) | 3,186(20.8) | 4,699(28.8) |
| **Vital capacity (ml)** | *P*5<0.001^#^, *P*6<0.001^#^, *P*7<0.001^#^, *P*8<0.001^#^, *P*9<0.001^#^, *P*10<0.001^#^ | | | | |
| **Standing long jump (cm)** | *P*5<0.001^#^, *P*6<0.001^#^, *P*7<0.001^#^, *P*8<0.001^#^, *P*9<0.001^#^, *P*10<0.001^#^ | | | | |
| **50-m dash (s)** | *P*5<0.001^#^, *P*6<0.001^#^, *P*7<0.001^#^, *P*8<0.001^#^, *P*9<0.001^#^, *P*10<0.001^#^ | | | | |
| **Total score** | *P*5<0.001^#^, *P*6<0.001^#^, *P*7<0.001^#^, *P*8<0.001^#^, *P*9<0.001^#^, *P*10<0.001^#^ | | | | |

**Note:** cm, centimeter; kg, kilogram; kg/m^2^ , kilogram/meter^2^ ; ml, milliliter; s, second.

^*^*P*<0.05,^#^*P*<0.001.

*P*1, *P*2, *P*3, *P*4, mean the p-values of Kruskal-Wallis H test between 2019-2020, 2020-2021, 2021-2022, and 2022-2023.

*P*5, *P*6, *P*7, *P*8, *P*9, *P*10 mean the p-values of Kruskal-Wallis H test between 2019-2021, 2019-2022, 2019-2023, 2020-2022, 2020-2023 and 2021-2023.

| **Table S2 Pairwise comparisons of physical fitness indicators from 2019 to 2023 by bonferroni method** | | | | | | | |
| --- | --- | --- | --- | --- | --- | --- | --- |
| **Gender** | **Items** | **A-C** | **A-D** | **A-E** | **B-D** | **B-E** | **C-E** |
| Male | Height (cm) | <0.001^#^ | <0.001^#^ | <0.001^#^ | <0.001^#^ | <0.001^#^ | <0.001^#^ |
|  | Weight (kg) | <0.001^#^ | <0.001^#^ | <0.001^#^ | <0.001^#^ | <0.001^#^ | <0.001^#^ |
|  | BMI (kg/m^2^) | 0.77 | <0.001^#^ | <0.001^#^ | <0.001^#^ | <0.001^#^ | <0.001^#^ |
|  | Vital capacity (ml) | 0.18 | <0.001^#^ | <0.001^#^ | <0.001^#^ | <0.001^#^ | <0.001^#^ |
|  | Standing long jump (cm) | 0.001* | <0.001^#^ | <0.001^#^ | <0.001^#^ | <0.001^#^ | <0.001^#^ |
|  | Sit-and-reach (cm) | 0.01* | <0.001^#^ | <0.001^#^ | <0.001^#^ | <0.001^#^ | <0.001^#^ |
|  | 50-m dash (s) | <0.001^#^ | <0.001^#^ | <0.001^#^ | <0.001^#^ | <0.001^#^ | <0.001^#^ |
|  | Pull-ups (count) | <0.001^#^ | <0.001^#^ | <0.001^#^ | <0.001^#^ | <0.001^#^ | <0.001^#^ |
|  | 1000 m (s) | <0.001^#^ | <0.001^#^ | 1 | <0.001^#^ | <0.001^#^ | <0.001^#^ |
|  | Total score | <0.001^#^ | <0.001^#^ | <0.001^#^ | <0.001^#^ | <0.001^#^ | <0.001^#^ |
| Female | Height (cm) | <0.001^#^ | <0.001^#^ | <0.001^#^ | <0.001^#^ | <0.001^#^ | 0.006* |
|  | Weight (kg) | 0.028* | <0.001^#^ | <0.001^#^ | <0.001^#^ | <0.001^#^ | <0.001^#^ |
|  | BMI (kg/m^2^) | <0.001^#^ | <0.001^#^ | <0.001^#^ | <0.001^#^ | <0.001^#^ | <0.001^#^ |
|  | Vital capacity (ml) | <0.001^#^ | <0.001^#^ | <0.001^#^ | <0.001^#^ | <0.001^#^ | <0.001^#^ |
|  | Standing long jump (cm) | <0.001^#^ | <0.001^#^ | <0.001^#^ | <0.001^#^ | <0.001^#^ | <0.001^#^ |
|  | Sit-and-reach (cm) | 0.001* | <0.001^#^ | <0.001^#^ | <0.001^#^ | <0.001^#^ | <0.001^#^ |
|  | 50-m dash (s) | <0.001^#^ | <0.001^#^ | <0.001^#^ | <0.001^#^ | <0.001^#^ | <0.001^#^ |
|  | Sit-ups (count) | <0.001^#^ | <0.001^#^ | <0.001^#^ | <0.001^#^ | <0.001^#^ | <0.001^#^ |
|  | 800 m (s) | <0.001^#^ | 0.43 | <0.001^#^ | <0.001^#^ | <0.001^#^ | <0.001^#^ |
|  | Total score | <0.001^#^ | <0.001^#^ | <0.001^#^ | <0.001^#^ | <0.001^#^ | 1 |

Note: A-B means the *P*-values of Kruskal-Wallis H test between 2019-2020 (for example); ^*^*p*<0.05, ^#^*p*<0.001

| **Table S3 Detailed data (Total score, Mean**±**SD) of four levels of BMI in Figure 3** | | | | | |
| --- | --- | --- | --- | --- | --- |
| **BMI** | **Y2019** | **Y2020** | **Y2021** | **Y2022** | **Y2023** |
| underweight | 66.9±7.7 | 67.1±6.9 | 70.5±7.2 | 71.4±7.5 | 73.1±9.2 |
| normal weight | 71.4±7.4 | 71.1±6.7 | 74.3±7.1 | 76.6±8.1 | 76.3±8.9 |
| overweight | 65.4±7.9 | 64.4±7.6 | 67.8±7.2 | 70.8±8.4 | 71.7±9.8 |
| obesity | 58.7±9.3 | 59.8±8.5 | 61.0±7.9 | 61.5±9.7 | 64.5±13.7 |
| Total score | 69.9±8.2 | 69.5±7.6 | 72.6±7.9 | 73.9±9.5 | 73.9±10.4 |

| **Table S4 Detailed data (N+%) of four levels of BMI in Figure 4 by gender** | | | | | |
| --- | --- | --- | --- | --- | --- |
| **BMI** | **Y2019** | **Y2020** | **Y2021** | **Y2022** | **Y2023** |
| **Underweight** | 6.71 | 8.11 | 7.47 | 3.86 | 3.25 |
| underweight for males | 3.05 | 3.03 | 3.17 | 1.89 | 1.79 |
| underweight for females | 3.66 | 5.09 | 4.31 | 1.98 | 1.46 |
| **Normal weight** | 77.25 | 76.18 | 76.45 | 68.00 | 63.72 |
| normal for males | 28.31 | 29.22 | 29.96 | 28.25 | 26.35 |
| normal for females | 48.94 | 46.94 | 45.49 | 39.75 | 37.36 |
| **Overweight** | 11.73 | 10.96 | 12.85 | 19.43 | 22.43 |
| overweight for males | 7.34 | 7.53 | 8.68 | 11.69 | 13.60 |
| overweight for females | 4.40 | 3.43 | 4.17 | 7.74 | 8.82 |
| **Obesity** | 4.30 | 4.75 | 4.23 | 8.71 | 10.61 |
| obesity for males | 2.95 | 3.19 | 3.04 | 5.83 | 7.22 |
| obesity for females | 1.36 | 1.56 | 1.19 | 2.88 | 3.38 |
